# Neurogenic thoracic outlet syndrome: a review of diagnostic and therapeutic strategies

**DOI:** 10.3389/fneur.2026.1932666

**Published:** 2026-10-06

**Authors:** Guang-Yao Li, Ming-Qiang Xue, Jing-Wei Wang, Ke Sha

**Affiliations:** 1 Department of Orthopedic Trauma and Hand Surgery, The First Affiliated Hospital of Guangxi Medical University, Nanning, Guangxi, China; 2 Research Centre for Regenerative Medicine, Guangxi Medical University, Nanning, Guangxi, China; 3 Guangxi Key Laboratory of Regenerative Medicine, Guangxi Medical University, Nanning, Guangxi, China

**Keywords:** diagnosis, outcome, surgery, thoracic outlet syndrome, treatment

## Abstract

The diagnosis and treatment of neurogenic thoracic outlet syndrome (nTOS) remain challenging. Although consensus-based clinical frameworks exist, they lack robust externa validation. This study conducts a review of nTOS publications in the PubMed, Embase, and Web of Science databases, with a search cutoff date of December 2025. Current evidence suggests that the incidence of nTOS may be underestimated. Diagnosis should be based on a comprehensive evaluation, including medical history, systematic physical examination, ancillary examinations, diagnostic muscle block, and exclusion of other possible diseases. Treatment should be carried out in a phased manner. For mild cases, nonsurgical treatment is preferred. For patients who experience progressive neurological dysfunction, muscle atrophy, or failure of conservative treatment, timely surgery should be considered. Surgical decompression mainly involves the supraclavicular or axillary approaches. Surgical techniques include first rib resection, rib-sparing scalenectomy, pectoralis minor release, cervical rib resection. However, the optimal treatment remains controversial due to the limited availability of high-quality evidence. Future research should focus on elucidating the pathophysiology of nTOS, improving diagnostic accuracy through technological innovation, and optimizing treatment protocols through prospective multicenter studies.

## Introduction

Thoracic outlet syndrome (TOS) is classified into neurogenic TOS (nTOS), venous TOS (vTOS), and arterial TOS (aTOS) subtypes. nTOS is the most common type, accounting for 90–95% of cases, with diverse clinical manifestations and complex pathophysiology (1). Epidemiologically, nTOS has a reported annual incidence of 2–3 cases per 100,000 individuals and a prevalence of 10 cases per 100,000 individuals, although these figures vary substantially across studies (2, 3). These results suggest that the actual burden of nTOS may be underestimated. Consequently, nTOS is still a challenge across orthopedics, cardiovascular thoracic surgery, plastic surgery, and neurology (4–6).

On the other hand, nTOS as an independent research topic has gained increasing attention (Figure 1). Goeteyn et al. (7) conducted a randomized controlled trial (RCT) comparing nTOS decompression surgery with conservative treatment. They found that the functional improvement in the surgical group was significantly greater than that in the conservative treatment group at 3 months postoperatively. However, after patients in the conservative treatment group received surgery and were followed for an additional 3 months, the difference between the two groups was no longer statistically significant. Derdiyok and Temel (8) reported the outcomes of 13 nTOS patients who underwent first rib resection (FRR). After an average follow-up of 19 ± 13 months, only one patient had mild symptoms, while all others achieved complete recovery. Kakamad (9) further compared the efficacy of FRR with first-rib avulsion, suggesting that avulsion may be a feasible alternative to resection, providing comparable results with less invasiveness. These studies demonstrate that nTOS patients can benefit from surgical decompression and that outcomes depend on accurate diagnosis, timely intervention, and appropriate treatment. Although consensus-based diagnostic and therapeutic frameworks are available for nTOS, no gold-standard exists for clinical management. Therefore, establishing a standardized framework for diagnosis and treatment is a crucial need. In this context, this study reviews the publications on nTOS, aiming to translate existing research findings into valuable strategies.

**Figure 1:**
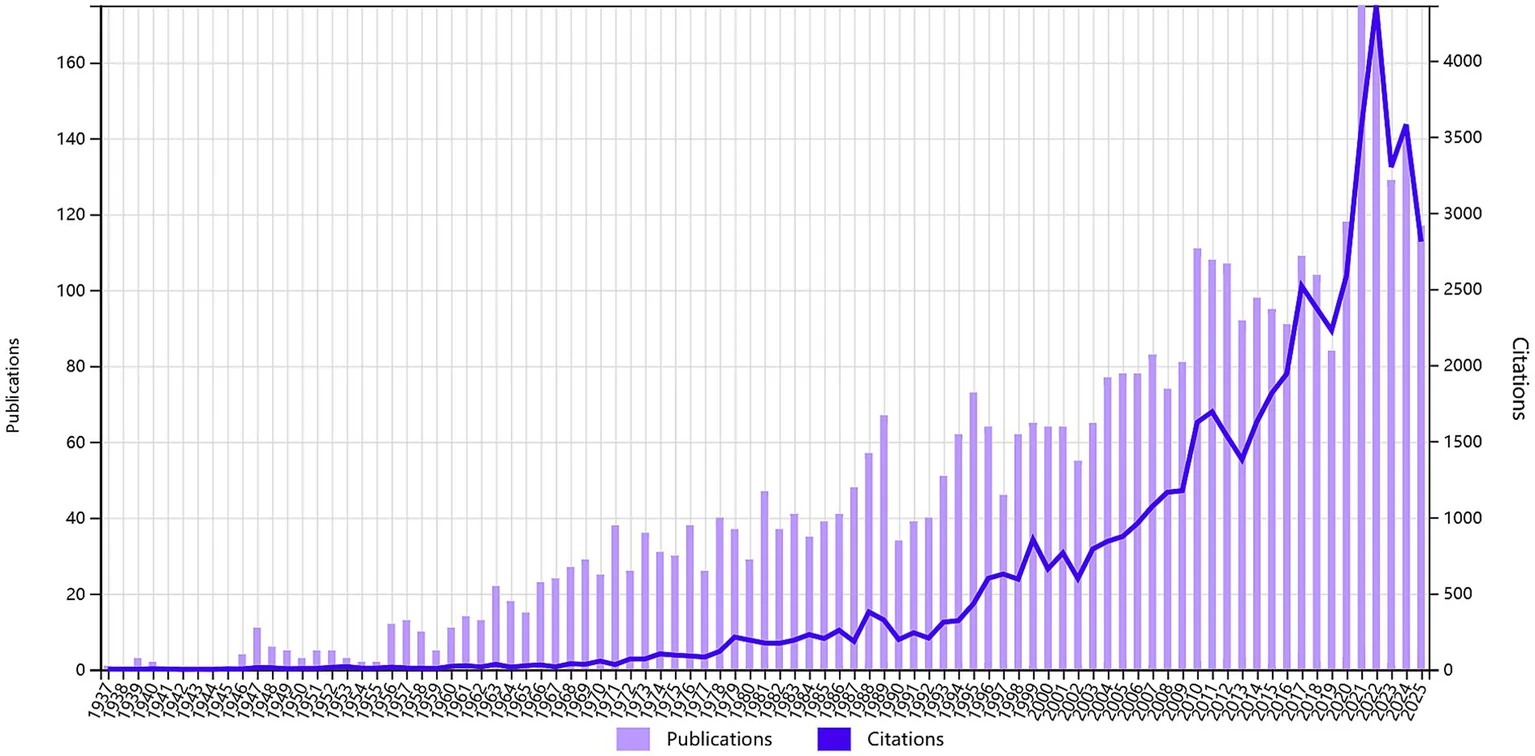
Publication and citation trends in neurogenic thoracic outlet syndrome research. (The figure was created through Webscience.com).

## Methods

This study adopted a structured narrative review approach to search for nTOS articles published up to December 2025, covering the PubMed, Embase, and Web of Science databases. The search strategy was designed according to systematic review standards, including peer-reviewed articles and manual screening of reference lists from relevant publications. The search terms combined “nTOS” with keywords such as “epidemiology,” “incidence,” “prevalence,” “imaging,” “diagnosis,” “conservative treatment,” “physical therapy,” “NSAIDs,” “surgery,” and “RCT.” Studies meeting the inclusion criteria were identified by screening the titles, abstracts, and full texts.

### Pathogenic factors

The thoracic outlet is an anatomical passageway between the lower neck and the axilla through which the neurovascular structures of the upper limb pass (10). It is bordered by the clavicle, the first rib, and the scalene muscles, and includes several potential compression sites, such as the interscalene triangle, costoclavicular space, and subcoracoid space (Figure 2). Narrowing or dynamic compression within these spaces can affect the brachial plexus (BP), resulting in nTOS. The pathogenesis of nTOS involves several key mechanisms:

**Figure 2:**
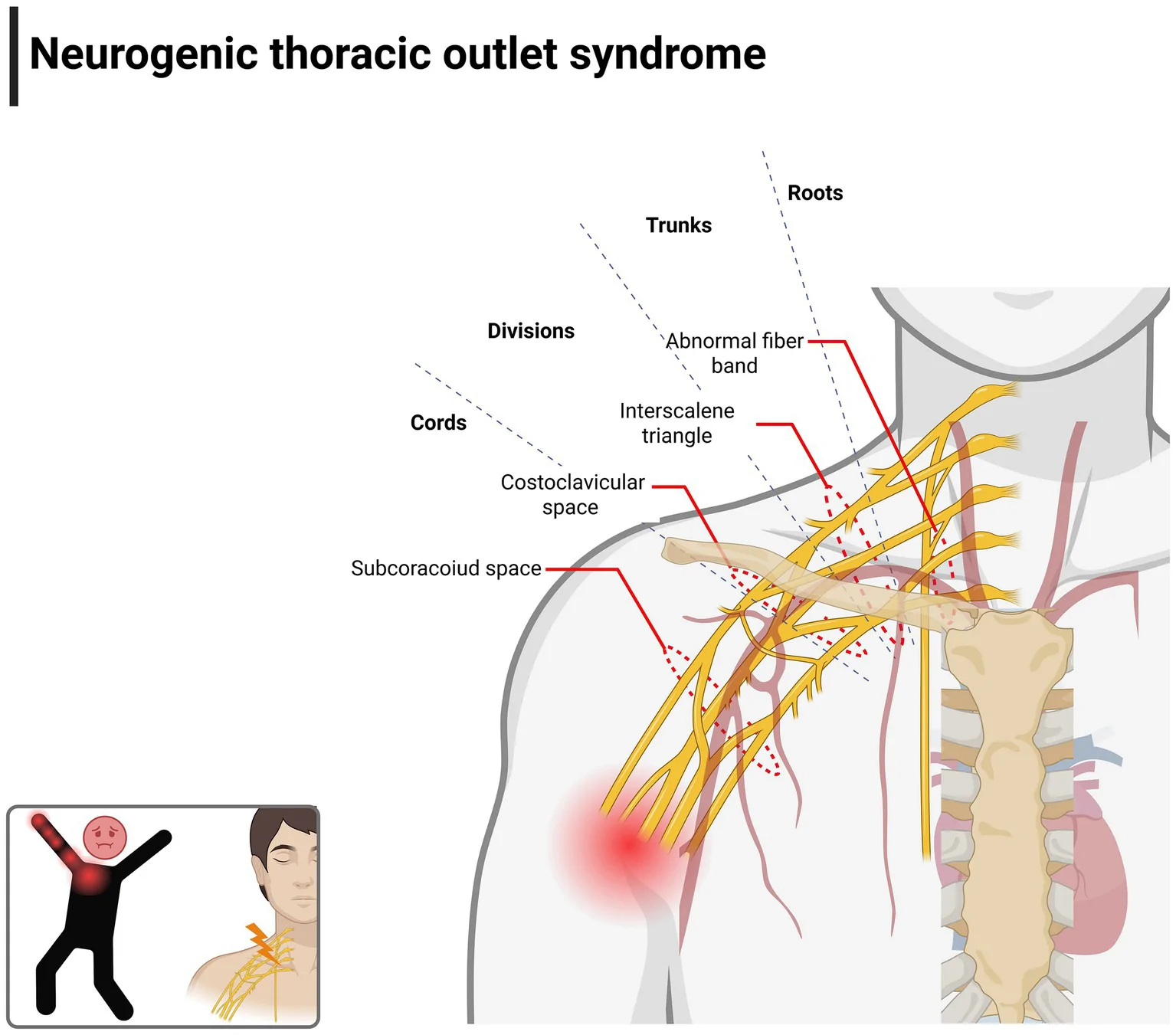
Neurogenic thoracic outlet syndrome (nTOS). nTOS arises when the brachial plexus is compressed by the clavicle, surrounding musculature, or abnormal fibromuscular bands, which produces symptoms of pain, paresthesia, and muscle weakness that radiate to the upper back, neck, shoulder, arm and hand. (The figure was created through Biorender.com).

#### Congenital factors

Congenital factors include both osseous and soft tissue abnormalities that may directly compress or irritate the BP. Osseous anomalies include cervical ribs, elongated C7 transverse processes, and first-rib anomalies, whereas soft tissue abnormalities involve anomalous scalene muscles and fibromuscular bands (4). Early studies focused on osseous abnormalities (5). However, after observing the persistence or recurrence of symptoms following bone resection, surgeons gradually shifted their focus to soft tissue abnormalities (11). In this regard, Roos (12) systematically described various abnormal fibromuscular bands associated with TOS, laying an important pathological foundation. The scalene anomalies include scalenus minimus and scalenus pleuralis variants which may cause compression within the thoracic outlet. Such compression can be spontaneous intrinsic compression, or secondary occurring after the addition of trauma. Trauma, a well-recognized etiologic factor, is discussed further below.

#### Traumatic factors

Common traumatic causes include whiplash injuries and fractures of the clavicle or first rib. These injuries may directly damage the BP or cause secondary compression through hemorrhage, edema, fibrosis, scar contracture, or malunion following fracture (4). A history of neck or shoulder trauma is frequently reported in nTOS patients, with some surgical series reporting rates of approximately 80–86% (13, 14); however, these proportions vary considerably across studies and should not be generalized to all nTOS patients.

#### Functional factors

Functional factors are recognized as important contributors to nTOS. Higher-risk populations include baseball pitchers, swimmers, industrial workers, military personnel, and office workers (15). Common triggers include prolonged poor posture, repetitive overhead activity, and high-intensity weight training for the upper body. These activities may lead to hypertrophy, spasm, hypertonicity, or adaptive shortening of the anterior and middle scalene muscles as well as the pectoralis minor. Consequently, these changes may dynamically narrow the space of the thoracic outlet, resulting in nTOS (16, 17).

Notably, the severity of symptoms may be influenced by pain sensitization, sleep disturbances, anxiety, and other psychosocial factors. These factors should be regarded as modifiers of the symptom burden rather than independent diagnostic criteria. When symptoms such as dizziness, tinnitus, Raynaud-like color changes, or diffuse subjective discomfort predominate, particularly in the absence of objective neurological or vascular abnormalities, clinicians should broaden the differential diagnosis rather than attributing all symptoms to nTOS-related autonomic or sympathetic disorders.

### Diagnosis

#### History-taking

The medical history should document potential triggers, initial symptoms, symptom characteristics, disease progression, and relevant demographic, occupational, and upper limb activity information. Clinicians should have a certain ability to distinguish the “authenticity” of medical history, rather than passively accepting the patient’s complaints. This is particularly important when evaluating populations such as manual laborers, as they are more likely to overlook initial symptoms. Muscle weakness may become their chief complaint, causing them to unconsciously overlook the development of initial symptoms. This phenomenon easily leads clinicians to fall into the “chief-complaint trap.” Therefore, it is crucial to reconstruct the disease progression by clarifying the timing and characteristics of the initial symptoms, including fatigue, sensory abnormalities, and pain. If the patient reports a history of upper respiratory infection or vaccination, followed by acute severe pain in the shoulder or upper arm, then a noticeable decline in muscle strength with gradual partial recovery, the diagnosis is more likely to be brachial plexus neuritis (BPN). In contrast, the typical symptoms of nTOS are initially mild and related to limb activity, and then progressively worsen over time (18, 19).

#### Physical examination

Physical examination should follow a standardized sequence: inspection, palpation, movement assessment, neurological evaluation, and provocative tests. The patient should sufficiently expose the cervicoscapular region and upper limbs for complete inspection. Inspection should focus on asymmetry, scapular dyskinesis, clavicular abnormality, and muscle atrophy in the distal upper limb (20). Spontaneous fasciculations, tremor, or other involuntary movements should also be noted, as these signs may suggest central nervous system pathology.

Palpation should assess the interscalene triangle, pectoralis minor, and scapular regions for tenderness, and percussion should be performed to elicit Tinel’s sign to localize potential nerve entrapment sites. Then active motion of the shoulder and neck should be evaluated. Movements such as abduction, elevation, and overhead activity of the affected limb should be performed to determine whether they provoke or exacerbate symptoms, thereby clarifying the relationship between posture, movement, and neurological signs.

Neurological examination is the core of physical assessment, encompassing muscle strength and tone, tendon reflexes, and sensory function. In typical nTOS, compression of the lower trunk of BP usually produces a T1-predominant, C8-associated pattern, with weakness or atrophy of the intrinsic hand muscles (21). In some cases, weakness may also involve C8-innervated forearm or hand muscles, whereas upper-trunk involvement is uncommon and should prompt careful reassessment of the diagnosis. The presence of hypertonia, hyperreflexia, Babinski signs, or other upper motor neuron signs should raise suspicion of cervical myelopathy or other central nervous system disorders. Grip strength testing is a useful tool in the evaluation of nTOS. The Jamar dynamometer is easy to use and has good repeatability, making it suitable for outpatient assessment (22). Serial assessments of grip strength before and after treatment can help evaluate functional status and treatment response, but they should not be interpreted as a standalone measure of disease severity.

Sensory examination should cover the entire upper limb, and may be extended to the neck or trunk when central or spinal cord disease is suspected. In unilateral cases, sensory findings can be compared with the contralateral limb; in bilateral cases, comparison with normally sensate trunk regions may be necessary (1). Characteristic sensory abnormalities in nTOS involve the C8–T1 distribution, especially the medial antebrachial cutaneous territory and the ulnar side of the hand, with hypoesthesia being more common than hyperesthesia. Sensory abnormalities that are disproportionate to, or dissociated from, progressive motor weakness should broaden the differential diagnosis rather than being attributed to nTOS.

#### Provocative tests

Provocative tests, including the Adson, Halsted, Wright, and Roos/EAST tests, have limited diagnostic utility (23, 24). According to Nord et al. (25), the false-positive rates of the Roos/EAST, Adson, Halsted, and supraclavicular compression tests in the general population are approximately 47, 9–20%, 16, and 30%, respectively. Whereas in patients with carpal tunnel syndrome, these false positive rates increase to 77, 42–45%, 48, and 61%, respectively. Furthermore, 56% of healthy participants and 94% of patients with carpal tunnel syndrome had at least one positive result in the provocative tests. Despite these limitations, the presence of multiple positive findings may enhance the diagnostic credibility. Importantly, the provocative test results should be interpreted with the medical history, physical examination, and ancillary examinations.

#### Diagnostic muscle block

Diagnostic muscle block can aid in diagnosing nTOS and predicting response to surgical decompression (1). A systematic review of 12 studies involving 950 patients reported that the combined sensitivity of scalene and pectoralis minor muscle blocks for diagnosing nTOS was 87%, with a specificity of 34% (26), showing high sensitivity with poor specificity. Image-guided injection not only improves accuracy in localizing target muscles but also reduces the risk of complications such as Horner’s syndrome, hoarseness, and swallowing issues (27–29). Additionally, Gillette et al. (30) reported on 63 patients who underwent preoperative diagnostic scalene muscle block, of whom 61 cases showed positive response. Among the 60 patients available for postoperative follow-up, 39 achieved complete symptom relief, 20 had partial improvement, and 1 showed no improvement. Therefore, diagnostic muscle block should be regarded as an important component of the clinical management, rather than as a standalone decision-making tool.

#### Ancillary examinations

Ancillary examinations include imaging tests, electrophysiological studies, and ultrasound. Each of them has its advantages and limitations. Magnetic resonance imaging (MRI), particularly MR neurography, can help detect BP compression, soft tissue abnormalities, and space-occupying lesions. However, it has limitations in assessing osseous structures (31). In contrast, computed tomography (CT) and X-ray are useful for identifying osseous abnormalities, such as cervical ribs, elongated C7 transverse processes, and first-rib or clavicular abnormalities, but they are unable to provide detailed evaluation about the soft tissues (10, 32). Electrophysiological studies can reveal nerve dysfunction and help exclude cervical radiculopathy and distal entrapment neuropathies. Nevertheless, their sensitivity is poor in early or mild cases, and normal neurophysiologic results do not rule out nTOS (33). High-resolution neuromuscular ultrasonography allows us to dynamically observe the morphology and trajectory of the BP, and its relationship with adjacent tissues (34, 35). However, its accuracy is heavily operator-dependent, and it has limited ability to visualize deep structures such as the T1 nerve root. Therefore, ancillary examinations should be selectively utilized to facilitate comprehensive clinical judgment according to individual patient conditions.

In summary, the diagnosis of nTOS should be based on a comprehensive assessment and multidisciplinary collaboration. Figure 3 presents an effective diagnostic strategy.

**Figure 3:**
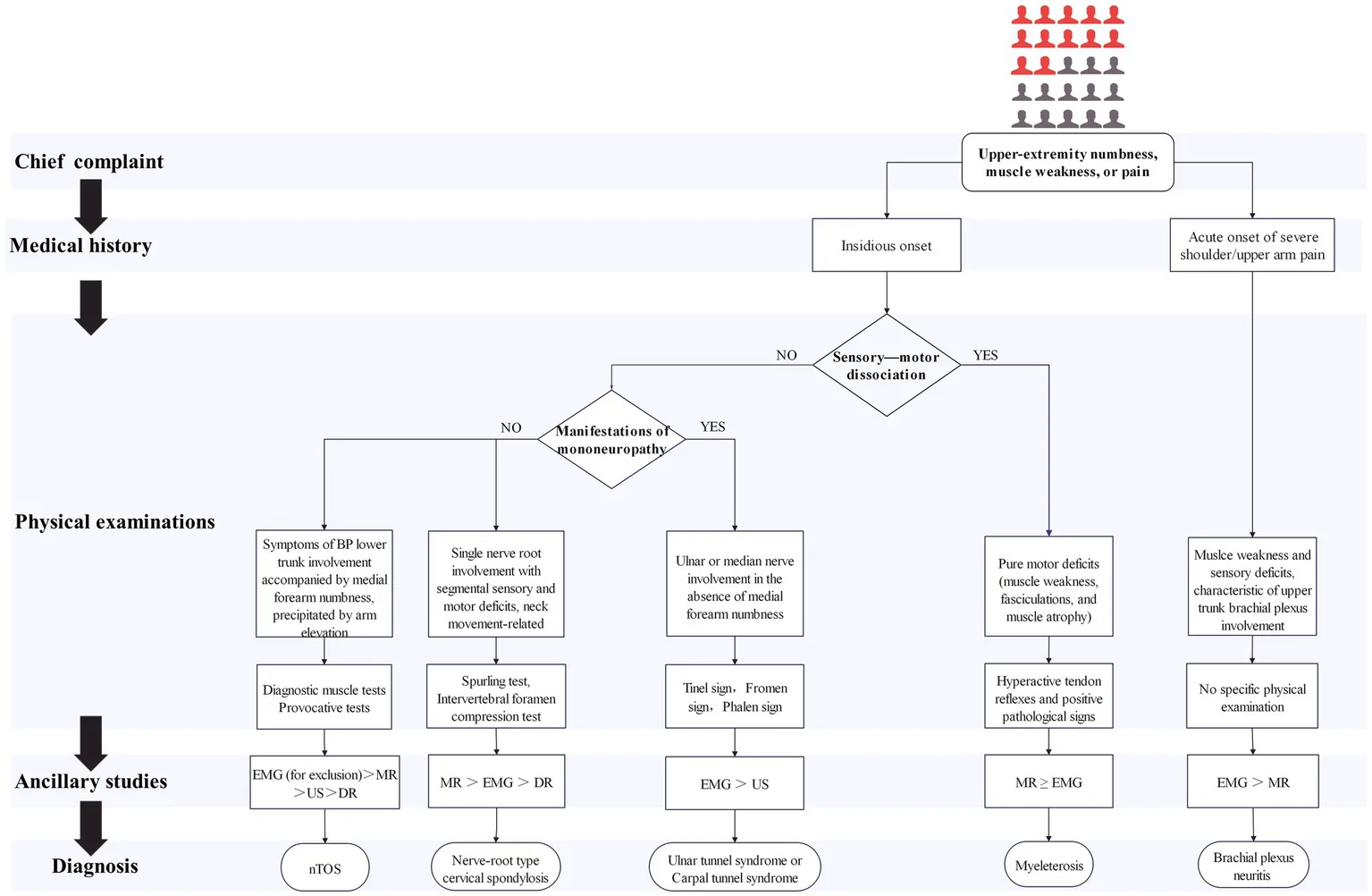
Differential diagnostic flowchart for neurogenic thoracic outlet syndrome and other upper-extremity neuropathic disorders.

### Treatment

#### Nonsurgical treatment

For early or mild cases, nonsurgical treatment is recommended and consists of patient education, physical therapy, botulinum toxin (BTX) injection, and cognitive behavioral therapy (36). Physical therapy is preferred, with the aim of relieving symptoms by restoring muscular balance in the neck and shoulder. Common methods include: (1) correcting postural abnormalities; (2) targeted stretching to relieve muscle tension and fatigue; (3) strengthening health education on correct sitting, lying, and walking postures to high-risk groups and suspected patients. The reported improvement rate of physical therapy is approximately 27 to 59%, but there is a lack of RCTs to confirm its long-term efficacy (37, 38). BTX injection has been reported to provide short-term improvement in some patients and help identify those likely to respond favorably to surgical decompression (15, 39). However, a double-blind controlled trial found no significant difference compared with placebo, and long-term follow-up data are insufficient (40). Analgesics, muscle relaxants, and neurotrophic drugs may temporarily alleviate symptoms, but their effects are limited and vary across individuals (41). Moreover,

psychological support is important for patients with prominent anxiety, sleep disturbance, or other psychosocial symptoms.

The major limitation of studies on nonsurgical treatment is the heterogeneity among them, such as treatment methods, duration, and frequency (37), making it difficult for a pooled analysis. Therefore, establishing a standardized nonsurgical treatment strategy should receive more attention.

#### Surgery

Surgery should be considered when there is clear evidence of nerve compression, progressive neurological dysfunction, or unsatisfactory recovery after 3–6 months of nonsurgical treatment (33). Dengler et al. (37) reported that the efficacy rate of surgical decompression can reach up to 90%. Notably, the timing of surgery has a decisive impact on outcomes. Early decompression not only prevents further worsening of nerve compression but may also promote neurological recovery (42, 43). In contrast, delayed surgery, while alleviating symptoms, often cannot completely reverse the already occurred denervation changes, muscle weakness, or muscle atrophy (44).

The surgical techniques for nTOS are diverse (Figure 4). However, no conclusive evidence indicates that any one technique has an absolute advantage in efficacy (33). The open supraclavicular approach is generally recommended, as it allows complete observation of the key structures such as the BP, scalene muscles, and subclavian vessels, thereby aiding in thorough decompression. However, this approach carries the risk of injuring pleura, phrenic nerve, and thoracic duct (45). To minimize surgical trauma, various minimally invasive techniques have been developed, including endoscopic axillary approach, video-assisted thoracoscopic surgery, arthroscopy, and robot-assisted surgery (46–50). These approaches generally proceed in an inferior-to-superior direction: the pectoralis minor is released at first, then the first rib is resected, followed by resection of the anterior and middle scalene muscles. This procedure avoids the complex anatomical structures of the cervical region and lowers the risk of iatrogenic injury. Additionally, robotic-assisted technology provides higher operational precision, although it requires specialized equipment and a trained surgeon. The principal limitation of minimally invasive technologies is limited operative exposure, which may result in incomplete decompression. Hosseinian et al. (51) found that chronic pain occurred more frequently in the axillary approach group than in the supraclavicular approach group at six-months follow-up. And the comparison of long-term outcomes between different approaches requires further investigation.

**Figure 4:**
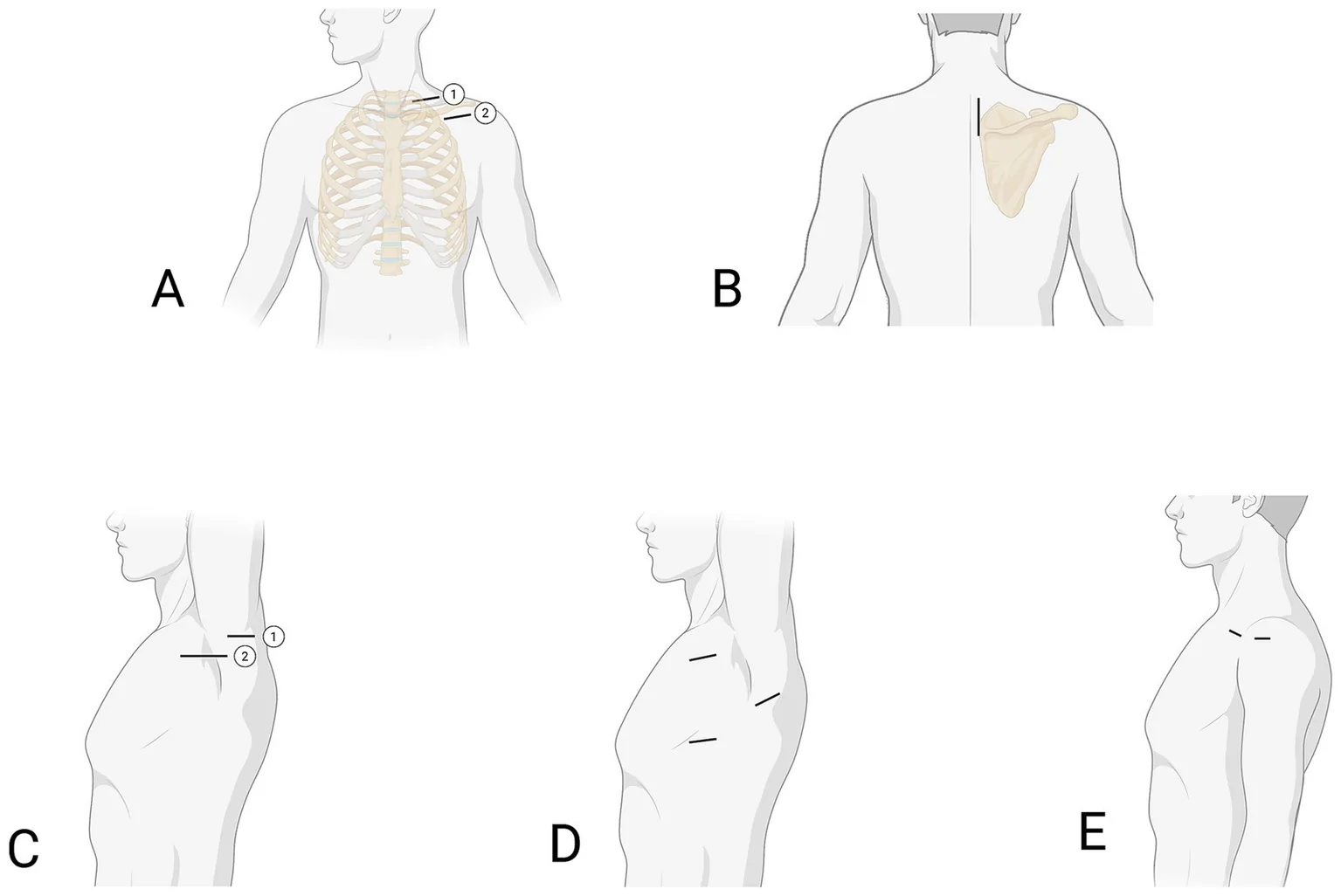
Surgical approaches for brachial plexus decompression in nTOS. **(A)** Supraclavicular approach (①), infraclavicular approach (①), and combined supraclavicular-infraclavicular approach (① + ②). **(B)** Posterior approach. **(C)** Axillary approach (②) and endoscopy-assisted axillary approach (① + ②). **(D)** Video-assisted thoracoscopic approach with multiple portals. **(E)** Arthroscopic pectoralis minor release and infraclavicular brachial plexus decompression via two portals. (The figure was created through Biorender.com).

Common surgical procedures include FRR, rib-sparing scalenectomy (RSS), cervical rib resection, pectoralis minor tenotomy or release, subclavius release, and excision of intraoperatively identified abnormal fibromuscular bands, scalenus minimus or scalenus pleuralis variants, or other compressive anatomical structures (52, 53). FRR has been widely adopted for nTOS, as it enlarges the space of the thoracic outlet and provides durable symptom relief (15, 54). Nuutinen et al. (55) reported favorable outcomes after transaxillary FRR, with excellent or good recovery in 81.5% of patients at long-term follow-up. However, the latest expert consensus does not explicitly recommend routine FRR (33). Furthermore, FRR carries risks such as pneumothorax, hemothorax, neurovascular injury, and residual rib stumps following incomplete resection. Endoscopically assisted transaxillary surgery also requires a long learning process. These factors may limit their application.

RSS gained increasing attention as a viable alternative to FRR in patients with nTOS. This interest stems from the recognition that BP compression may arise from pathological soft-tissue, including hypertrophy, spasm, fibrosis, or scarring of the anterior, middle or anomalous scalene muscles, rather than from osseous abnormalities alone. Ansari et al. (56) argued that RSS can achieve adequate BP decompression by resecting abnormal scalene muscles and fibromuscular bands while potentially reducing the morbidity and recovery burden associated with rib resection. In a long-term retrospective study of 94 patients with nTOS who underwent RSS, Ruopsa et al. (57) reported that 69% of patients experienced sustained benefit for a mean duration of 9.9 years. At a mean follow-up of 12.9 years, 82% of women and 57% of men rated their outcomes as excellent or good. Shekouhi et al. (58) further noted that RSS was associated with significantly better postoperative scores, although RSS and FRR could effectively improve symptoms. However, the available evidence on RSS is largely derived from retrospective studies. Therefore, intraoperative assessment following RSS, including passive upper-limb movement, may help determine whether first-rib-related compression exists and the necessity for further FRR.

Given these findings, it remains uncertain whether FRR or RSS should be preferred. Large-scale RCTs are warranted to compare their long-term efficacy, complication profiles, and reintervention rates. In summary, the choice between FRR and RSS should be individualized according to the presence of cervical or first-rib abnormalities, symptom severity, prior treatment history, surgeon expertise, institutional experience, availability of specialized equipment, and a balanced assessment of the advantages and risks of each procedure.

### Outcome assessment tools

A single pain score or subjective symptom description cannot fully capture the severity and functional recovery of nTOS. Therefore, a multidimensional assessment strategy should be employed to evaluate limb function. The Thoracic Outlet Syndrome Index (TOSI) is a comprehensive assessment tool for TOS that includes five dimensions: pain and physical symptoms, sports, work, lifestyle, and emotions (59, 60). The Disabilities of the Arm, Shoulder and Hand (DASH) questionnaire or QuickDASH can be used to assess the function of upper limbs (61), whereas the Cervical Brachial Symptom Questionnaire (CBSQ) reflects symptoms of the neck and arm (62). The Brachial Assessment Tool (BrAT), a validated tool, has good applicability in recording functional score of BP (63, 64). The 12-Item Short Form Health Survey (SF-12) can be used to evaluate health-related quality of life across physical and mental health domains (65). In addition to patient-reported outcome measures, objective indicators such as grip strength and pinch strength may be incorporated into pre- and post-treatment assessments. Combining subjective and objective measures may improve the reliability of efficacy assessment and the comparability of outcomes across studies.

### Future challenges

The pathogenesis of nTOS is complex, highlighting the need for further research into its cellular and molecular mechanisms. Highly sensitive and specific diagnostic indicators for nTOS remain lacking. Future studies should therefore focus on optimizing neuroimaging techniques and identifying valid biomarkers. More RCTs are required to generate high-level evidence for refining clinical strategies. Notably, systematic analysis of existing outcome-assessment instruments is warranted to refine or develop nTOS-specific evaluation tools.

## Conclusion

The pathogenesis of nTOS is complex. The diagnosis of nTOS should be based on a comprehensive assessment of the medical history, physical examination, provocative tests, diagnostic muscle block, and ancillary examinations. For mild or early cases, nonsurgical treatment is preferred. For patients with progressive neurological dysfunction, unsatisfactory recovery after conservative treatment, or clear signs of BP compression, timely surgery should be considered. The surgery plan should be formulated according to the specific condition of each patient. Robotic or endoscopic-assisted techniques may improve surgical precision, but they carry a risk of incomplete decompression. It is imperative to conduct high-quality studies with standard assessment tools and long-term follow-up to generate more valuable evidence.
